# Remote Evaluation of Sleep and Circadian Rhythms in Older Adults With Mild Cognitive Impairment and Dementia: Protocol for a Feasibility and Acceptability Mixed Methods Study

**DOI:** 10.2196/52652

**Published:** 2024-03-22

**Authors:** Victoria Grace Gabb, Jonathan Blackman, Hamish Duncan Morrison, Bijetri Biswas, Haoxuan Li, Nicholas Turner, Georgina M Russell, Rosemary Greenwood, Amy Jolly, William Trender, Adam Hampshire, Alan Whone, Elizabeth Coulthard

**Affiliations:** 1 Bristol Medical School University of Bristol Bristol United Kingdom; 2 Neurology Department Bristol Brain Centre, North Bristol NHS Trust Bristol United Kingdom; 3 King’s College Hospital King's College Hospital NHS Foundation Trust London United Kingdom; 4 Bristol Royal Infirmary University Hospitals Bristol and Weston NHS Foundation Trust Bristol United Kingdom; 5 Research & Innovation University Hospitals Bristol and Weston NHS Foundation Trust Bristol United Kingdom; 6 Department of Brain Sciences Faculty of Medicine Imperial College London London United Kingdom; 7 UCL Queen Square Institute of Neurology Faculty of Brain Sciences University College London London United Kingdom

**Keywords:** feasibility, sleep, mild cognitive impairment, dementia, Lewy body disease, Alzheimer disease, Parkinson, wearable devices, research, mobile phone, electroencephalography, accelerometery, mobile applications, application, app, cognitive, cognitive impairment, sleeping, sleep disturbance, risk factor, Alzheimer, wearable, wearables, acceptability, smart device

## Abstract

**Background:**

Sleep disturbances are a potentially modifiable risk factor for neurodegenerative dementia secondary to Alzheimer disease (AD) and Lewy body disease (LBD). Therefore, we need to identify the best methods to study sleep in this population.

**Objective:**

This study will assess the feasibility and acceptability of various wearable devices, smart devices, and remote study tasks in sleep and cognition research for people with AD and LBD.

**Methods:**

We will deliver a feasibility and acceptability study alongside a prospective observational cohort study assessing sleep and cognition longitudinally in the home environment. Adults aged older than 50 years who were diagnosed with mild to moderate dementia or mild cognitive impairment (MCI) due to probable AD or LBD and age-matched controls will be eligible. Exclusion criteria include lack of capacity to consent to research, other causes of MCI or dementia, and clinically significant sleep disorders. Participants will complete a cognitive assessment and questionnaires with a researcher and receive training and instructions for at-home study tasks across 8 weeks. At-home study tasks include remote sleep assessments using wearable devices (electroencephalography headband and actigraphy watch), app-based sleep diaries, online cognitive assessments, and saliva samples for melatonin- and cortisol-derived circadian markers. Feasibility outcomes will be assessed relating to recruitment and retention, data completeness, data quality, and support required. Feedback on acceptability and usability will be collected throughout the study period and end-of-study interviews will be analyzed using thematic analysis.

**Results:**

Recruitment started in February 2022. Data collection is ongoing, with final data expected in February 2024 and data analysis and publication of findings scheduled for the summer of 2024.

**Conclusions:**

This study will allow us to assess if remote testing using smart devices and wearable technology is a viable alternative to traditional sleep measurements, such as polysomnography and questionnaires, in older adults with and without MCI or dementia due to AD or LBD. Understanding participant experience and the barriers and facilitators to technology use for research purposes and remote research in this population will assist with the development of, recruitment to, and retention within future research projects studying sleep and cognition outside of the clinic or laboratory.

**International Registered Report Identifier (IRRID):**

DERR1-10.2196/52652

## Introduction

### Background

Dementia is the leading cause of death in the United Kingdom [1]. While the search continues for disease-modifying therapies, key research priorities include preventing, identifying, and reducing dementia risk and improving symptom burden and quality of life for patients with dementia and those who care for them [2]. With increasing evidence to support poor sleep as an important risk factor for dementia [3-5], sleep may offer an untapped opportunity in both reducing dementia incidence and improving quality of life for those with or at risk of developing dementia.

### Sleep and Dementia

Sleep is essential for optimal brain function and health [3]. Disrupted sleep and circadian rhythms are considered among the most debilitating symptoms in dementia, and increasing evidence suggests that sleep disturbances are a consequence of and contribute toward neurodegeneration underlying dementia including Alzheimer disease (AD) and Lewy body disease (LBD) [3,6].

Changes to sleep such as shorter total sleep time, more nocturnal awakenings, less time spent in deep slow-wave and rapid eye movement (REM) sleep, and a slight shift to earlier circadian rhythms are commonly observed as we age [7,8]. Most of these changes appear to stabilize around the seventh decade of life in healthy older adults [9]. In AD, sleep and circadian disturbances are more robust and severe than the changes seen in normal aging [10,11]. Some people with AD also experience a phenomenon known as “sundowning” (worsening of neuropsychiatric symptoms in the late afternoon or evening), which is thought to be in part caused by disturbances in circadian rhythms [12,13]. LBD is more typically associated with worse subjective sleep quality, REM sleep behavior disorder, sleep-related movement disorders such as restless legs syndrome, and higher levels of daytime sleepiness [5] compared to other dementias.

### Sleep as a Modifiable Risk Factor for Dementia

Individuals with sleep disorders (such as insomnia), sleep-disordered breathing, sleep-related movement disorders, circadian rhythm disorders, and poor quality or insufficient sleep are more likely to develop dementia later in life [14].

Mechanistically, chronic sleep deprivation and fragmentation are associated with various neurodegenerative processes including neuroinflammation [15], amyloid deposition [16,17], autophagy [15], tau phosphorylation [18], and hippocampal atrophy [19,20]. Sleep and circadian disturbances can precede cognitive and functional impairment, appearing in cognitively unimpaired older adults with AD biomarkers such as decreased cerebrospinal fluid amyloid-beta 42 and in mild cognitive impairment (MCI), and correlate with severity of cognitive impairment in AD [21-23]. Sleep disturbances also appear early in the disease course for LBD, particularly REM sleep behavior disorder [24,25]. Profiling sleep may offer noninvasive biomarkers for earlier diagnosis and staging, as well as targets for intervention to improve prognosis.

### Improving Quality of Life and Symptom Burden for People With Dementia

Sleep disturbances impact daily functioning, socialization, emotional well-being, and cognitive function in patients [26] and have a profound impact on caregivers [27]. Identifying targets for sleep interventions, whether tailored to an individual’s sleep profile or general advice given alongside dementia diagnosis or care, could help improve the quality of life for both persons with dementia and their caregivers. A single night of sleep deprivation disrupts cognitive performance [28], increases AD-related pathology such as amyloid burden [29], and reduces waste clearance in the brain [30], and improving sleep through treating sleep-disordered breathing has been associated with improvements in both neuropsychological assessments and blood biomarkers relating to AD in people with MCI [31]. Therefore, improving sleep could also benefit those with already established dementia and MCI, in addition to reducing incidence.

Further research is warranted to identify the most important sleep metrics and different sleep profiles in older adults with and without cognitive impairment to help identify targets for intervention [32].

### Measuring Sleep in Individuals With, or at Risk of, Dementia

Technological advancements in “wearables” (such as smartwatches and electroencephalography (EEG) headsets), “nearables” (such as a mattress or room sensors), and smartphones offer the unprecedented ability for tracking sleep at home for both consumers and researchers—to varying degrees of accuracy and accessibility [33,34]. The use of wearable technology is not new to sleep medicine or research; the current gold standard for sleep medicine is polysomnography (PSG) conducted under laboratory conditions, and wrist-worn actigraphy has been used for decades alongside paper-based sleep diaries to monitor rest and activity patterns typically over days or a couple of weeks [35]. However, most studies to date assessing sleep in individuals with MCI and early dementia have used questionnaires, with fewer studies adopting objective sleep technologies that could complement them such as actigraphy and EEG [32,36].

Alongside technological advancements, improved digital literacy and accessibility in older adults in recent years offer great promise for sleep research. A 2020 survey identified that 94.6% of 55-64 year olds, 85.5% of 65-74 year olds, and 54% of those aged 75 years and older had used the internet in the last 3 months [37]. Leveraging technology and remote assessments offers several potential benefits above PSG under laboratory conditions. PSG is often used across 1 or 2 nights, potentially leaving results vulnerable to the well-established “first-night effect,” which describes how sleep is quantitatively and qualitatively different during the first compared to subsequent night recordings. This has been observed to affect REM and non-REM sleep, awakenings, total sleep time, and subjective sleep quality [38]. PSG also limits mobility during the night. Conducting research in the home setting is more likely to capture naturalistic sleep as participants can largely continue their usual sleep-wake routines. Remote assessments enable longitudinal assessment, which may uncover natural night-to-night sleep variations. Newer sleep technologies enable the collection of both objective (via wearables and nearables) and subjective (via smartphone apps) sleep data that are considered the best practice to accurately capture sleep quality in older adults [39]. Subjective and objective sleep data may produce complementary or conflicting results [22] and allow multiple aspects of sleep (architecture vs experience) to be captured, enabling comprehensive profiling of sleep. Circadian and infradian rhythms may also be more accurately captured over a longer assessment period than is practical in a laboratory or outpatient setting, using actigraphy and repeated saliva samples that participants complete themselves. Finally, since participants do not need to attend sleep clinics, remote and technology-supported research may improve accessibility to research studies for those with less access to transport, reduce participant and study partner burden, and be more affordable, allowing for larger sample sizes.

However, before large-scale clinical trials and observational studies invest in and adopt technology- and home-based sleep measurements for dementia research, it is important to determine whether research conducted in this way is feasible and acceptable to older adults with and without cognitive impairment or dementia. People living with MCI and dementia experience changes in their communication or thinking, which may influence their experience of remote research, or they may have difficulty remembering to complete or understanding tasks without in-person support from a researcher; however, remote research may offer significant benefits including overcoming logistical issues typically faced in research and thus increase participation while reducing study burden [40]. The few studies that have addressed the feasibility of home-based sleep research and wearable technologies have often collected basic short-term feasibility data across only a few nights [41,42] and have typically required participants to be supported by a caregiver or care home staff [43,44]. Research is needed to see if community-dwelling participants with MCI and early dementia tolerate remote sleep and memory testing across an extended period of weeks or months (as would be expected in a clinical trial setting) and if they themselves can complete the study tasks. Caregivers or partners can provide important contributions to sleep and dementia research [45,46] but they often report poor quality sleep and high burden [47,48], and requiring a study partner may be a barrier to enrollment in research [49]. Independent (or minimally supported) involvement in research in milder stages of cognitive impairment may also positively acknowledge someone’s cognitive ability to engage in autonomous decision-making regarding their health [50].

Improving how we measure sleep and cognition in this population can deepen our understanding of the link between sleep and brain health, advice around sleep we give to patients, and improve monitoring in future interventional studies.

### Objectives

We will test the feasibility and acceptability of remote, in-home sleep and cognitive testing in a cohort of older adults with MCI or mild to moderate dementia due to AD or LBD and older adults without cognitive impairment. We hypothesize that using technology (wearable devices and smart devices) and remote study tasks will be well-tolerated by all study participants. Firstly, we will apply mixed methods to evaluate the feasibility and acceptability of remote study tasks based on the recruitment and retention of study participants, participant adherence to remote study tasks, data quality and completeness, and qualitative feedback on study tasks from participants. Secondly, we will explore whether sociodemographic or clinical variables explain any of the variability in feasibility and acceptability outcomes (whether someone is supported with tasks at home, cognitive impairment at baseline, and psychological variables at baseline such as apathy and anxiety). Thirdly, we will compare agreement on core sleep outcome measures (such as total sleep time, sleep efficiency, nocturnal awakenings, and sleep quality) across different measures. Finally, we will explore key themes in feedback from participants to identify strengths, limitations, and guidance for future sleep and remote-based research.

It is envisaged that these outcomes will be used to inform future research methodologies for both observational and interventional sleep research in older adults with and without dementia.

## Methods

### Study Design

This is a mixed methods study assessing the feasibility and acceptability of a novel combination of remote technology-supported sleep and cognitive assessments in older adults with and without cognitive impairment and dementia. The feasibility study is embedded within a prospective, longitudinal, and observational cohort study called the Remote Evaluation of Sleep to Enhance Understanding of Early Dementia (RESTED) study. Participants will complete baseline assessments and undergo remote sleep and cognitive assessments for a main study period of 8 weeks and a follow-up cognitive assessment at 6 months. Feasibility and acceptability will be assessed through the analysis of quantitative and qualitative data collected throughout the study and during the end-of-study interviews. Qualitative data will help to contextualize and enhance quantitative outcomes to deliver a more comprehensive analysis of the feasibility and acceptability [51].

### Setting

The study will be conducted at the Bristol Brain Centre, Southmead Hospital, within the North Bristol NHS (National Health Service) Trust and is sponsored by the University of Bristol. Baseline and follow-up assessments will be conducted at Southmead Hospital, remotely via phone or video call, or at the participant’s home. Participants will be asked to complete study activities from home, with visits from a researcher where needed, to deliver or collect study materials or provide support with study activities. Participants will be asked to complete a follow-up cognitive assessment at 6 months.

### Participants and Sample Size

Participants will be eligible if they are 50 years of age and older at consent, have full capacity to consent and are willing to adhere to study procedures, have Wi-Fi at home, and meet the criteria to fall into 1 of the 3 study arms: AD group, LBD group, and the control group.

For the AD group, participants will require a clinical diagnosis of MCI due to probable AD or mild AD dementia obtained from medical records. This may include participants with mixed dementia where AD is considered a significant component of clinical presentation.

For the LBD group, participants will require a clinical diagnosis of established or prodromal Parkinson disease dementia, dementia with Lewy bodies, MCI due to Parkinson disease, or MCI due to LBD obtained from medical records. This may include participants with mixed dementia where LBD is considered a significant component to clinical presentation.

For the control group, participants will confirm that they have no known cognitive impairment or neurodegenerative condition. Efforts will be made to match the AD and LBD cohorts on age and sex.

For all groups, participants with a clinically significant untreated sleep disorder predating or unrelated to a dementia diagnosis (such as narcolepsy or untreated sleep apnea), a severe medical or psychiatric comorbidity that may substantially impact sleep (such as refractory epilepsy), or a diagnosis of dementia other than AD or LBD will be excluded from the study.

Study participants will be recruited from cognitive and movement disorders clinics at the North Bristol NHS Trust, volunteer databases, and Join Dementia Research. The study is expected to be open to recruitment between February 2022 and June 2023 with a recruitment target of 75 participants (n=25 in each group). Prospective participants will be introduced to the study via a telephone call from the research team or during a meeting with their clinical team and provided with a digital or paper copy of the participant information sheet. Those who are interested in taking part will be invited to a screening visit for further discussion and, if agreeable, to provide consent. The participants will be asked if they would like to attend with a friend or relative, but we will not recruit formal study partners.

Following consent, participants will undergo a Montreal Cognitive Assessment (MoCA). Those scoring <11/30 will be withdrawn from the study as this would indicate more advanced cognitive impairment, unless in the opinion of the principal investigator that there is a mitigating factor impacting performance on the MoCA (such as prominent speech disorder), in which case they will be eligible to continue in the study.

### Outcome Measures

#### Brief Overview of the RESTED Study

Participants in the RESTED study will be asked to undergo screening and baseline assessments, including the MoCA, medical and clinical observations, questionnaires on sleep (Pittsburgh Sleep Quality Index [52], including responses from a cohabitant if available at the assessment, Epworth Sleepiness Scale [53], STOP (Snoring Tiredness Observed Pressure)-Bang Questionnaire [54], REM Sleep Behavior Disorder Single-Question Screen [55], Ultra-Short Version of the Munich ChronoType Questionnaire [56]), anxiety (Generalized Anxiety Disorder—7-item scale [57]), depression (Patient Health Questionnaire depression scale—8 [58]), and apathy (Apathy Evaluation Scale [59]). Participants will be asked to undergo blood biomarker testing for potential biomarkers of AD (amyloid beta 40 and 42, phosphorylated tau 181), neuroinflammation (glial fibrillary acidic protein), and neurodegeneration (neurofilament light chain) and overnight pulse oximetry using the Nonin 3150 WristOx_2_ to screen for obstructive sleep apnea (OSA), except where a diagnosis of OSA has already been given or assessment has been completed within 6 months of recruitment to the study. The main study period will involve 8 weeks of remote study tasks, such as sleep diaries (Consensus Sleep Diary—Main [60] with a bespoke additional question on comparison of the previous night to typical sleep), wrist-based actigraphy (Axivity AX3), and regular online cognitive tests (choice reaction time, digit span, and self-ordered search via Cognitron). Throughout the main study period, participants are supported by researchers via MyDignio, a patient-facing mobile platform designed specifically for delivering remote health care, using task reminders and checklists. During 1 of the 8 weeks, participants will undergo an “intensive week” consisting of daily browser-based cognitive testing, wearing a Dreem 2 EEG headband during sleep, saliva samples for dim light melatonin onset (evening) and cortisol awakening response analyses (morning), and verbal memory recall and recognition tasks via a video link with a researcher. Participants will also be asked to provide feedback on their experiences throughout the study. A subsample of participants will be invited to take part in an end-of-study interview. Finally, participants will be invited to a 6-month follow-up MoCA.

#### Feasibility Outcomes

**Figure 1:**
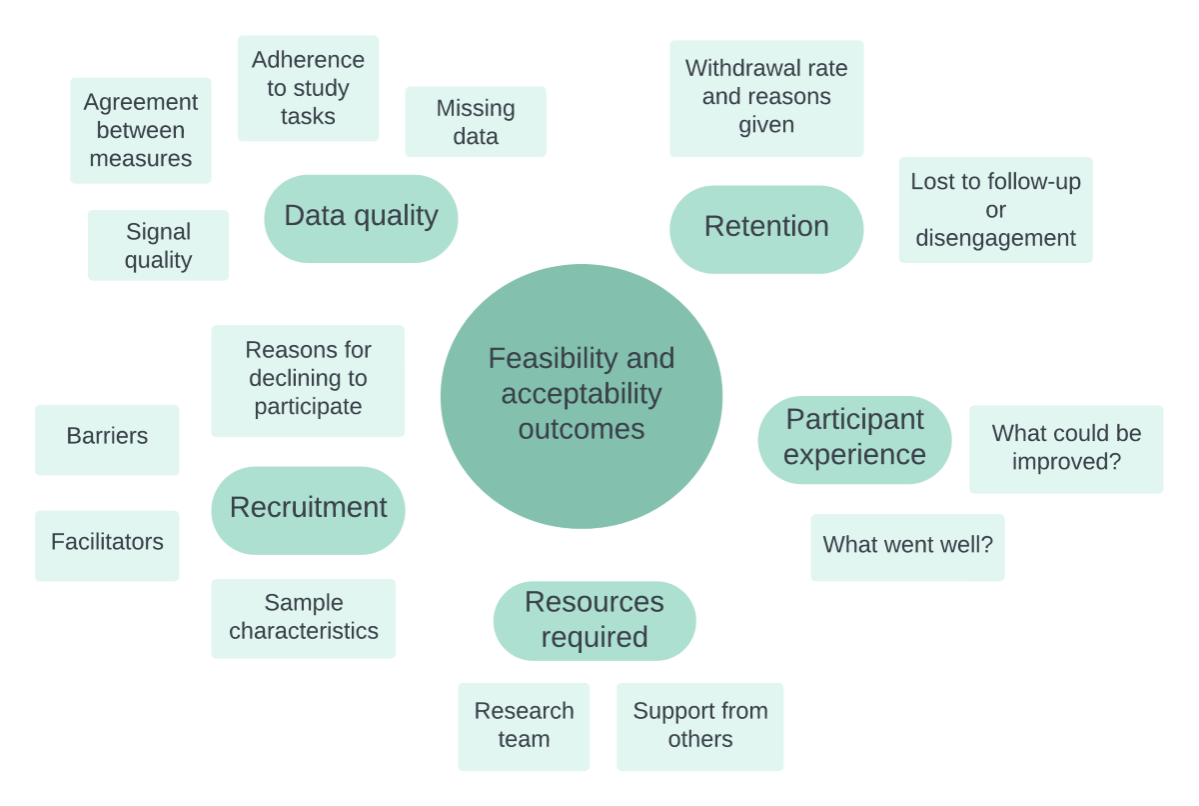
Conceptual map of feasibility and acceptability outcomes for the remote evaluation of sleep to enhance understanding of early dementia study. The core outcomes will be recruitment and retention, data quality, resources required, and participant experience.

The feasibility and acceptability outcomes will be predominantly based on data relating to recruitment and retention, the remote study tasks (Table 1), and participant feedback. The core outcomes of the study are outlined in Figure 1.

**Table 1:** Summary of data collection methods and frequency of remote home-based study tasks for the Remote Evaluation of Sleep to Enhance Understanding of Early Dementia study.

Remote study task	Method of data collection	Frequency of data collection	Duration of data collection
Sleep diary	App-based, via MyDignio	Daily	8 weeks
Actigraphy	Wrist-based actigraphy	Continuous	8 weeks
Remote browser-based cognitive tasks	Participants’ own device, via Cognitron website	Twice per week, then daily during intensive week	8 weeks
Recall and recognition tasks with a researcher	Videoconferencing software	Four brief tasks to complete across 4 separate days (<5 minutes each)	1 week (intensive week only)
Overnight electroencephalography (EEG)	Dreem EEG headband	Every night	1 week (intensive week only)
Saliva samples for cortisol awakening response	Saliva swabs	Three swabs (0, 30, and 60 minutes after awakening)	1 morning (intensive week only)
Saliva samples for dim light melatonin assay	Passive drool samples	Seven samples hourly starting from 5 hours before usual bedtime	1 evening (intensive week only)
Overnight pulse oximetry for sleep apnea screening	Pulse oximeter	Overnight	2 nights

#### Recruitment and Retention Rates

**Figure 2:**
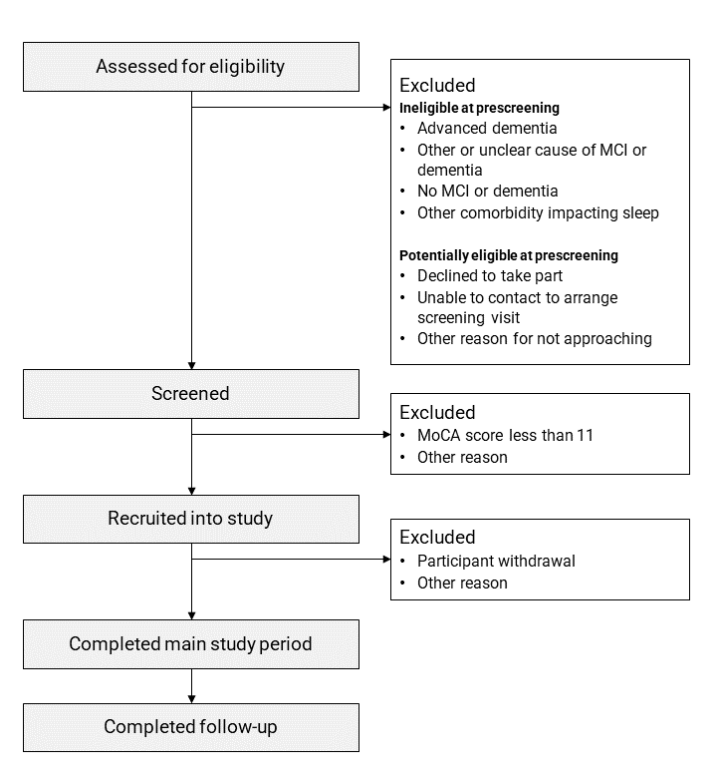
A template flowchart of participant flow through the RESTED study, based on STROBE (Strengthening the Reporting of Observational Studies in Epidemiology) guidelines. This flowchart will document participant recruitment and retention at each stage of the study. MCI: mild cognitive impairment; MoCA: Montreal Cognitive Assessment; RESTED: Remote Evaluation of Sleep to Enhance Understanding of Early Dementia.

Recruitment and retention to the study will be described and presented in a flowchart following Strengthening the Reporting of Observational Studies in Epidemiology (STROBE) guidelines (Figure 2). Key recruitment and retention outcomes will include the proportion of eligible patients who consent to take part in the study and the proportion of patients who withdraw from the study after consent. Reasons for ineligibility and nonparticipation at each stage (prescreening, screening, main study period, and follow-up), as well as barriers and facilitators to recruitment, will be summarized. Sample characteristics will be presented in tables.

#### Data Quality, Participant Adherence, and Data Completeness

The participants will be provided written and verbal guidance and reminders on how and when to complete each of the study tasks. Descriptive statistics on adherence and data completeness for each remote study task will be summarized (eg, the number of nights the EEG headband was worn and the number of completed sleep diaries). Reasons for incompleteness (eg, participant nonadherence and technical problems) and methods used to encourage or improve data completion (eg, reminders to complete tasks) will be described.

We will also assess the extent to which data appear to be valid and of sufficient quality for analysis of core sleep metrics (eg, for EEG data, this will include individual EEG channels and overall record quality metrics).

Where multiple sleep metrics are measured on a single night (eg, total sleep time via actigraphy, sleep diary, and EEG), an agreement between different measurement tools will be calculated (eg, Bland-Altman plot comparing sleep diary-adjusted actigraphy to EEG).

#### Resources

We will assess the amount of support and resources (eg, in-person visits, email, app-based, and telephone support) required from the research team to complete remote study tasks. Though the study will not require a study partner, we will also record whether participants perceive that they have access to support from outside of the study team (eg, family member, caregiver, or friend), whether this support is used, and what support is provided (eg, technical support and reminders).

#### Barriers, Facilitators, and Participant Feedback

We will review participant feedback expressed before, during, and at the end-of-study interviews to identify barriers and facilitators in (1) participating in remote sleep and cognitive research in general and (2) study-specific remote tasks.

The end-of-study interviews were designed and guided by the Capability Opportunity Motivation-Behavior (COM-B) system model of behavior, which suggests that capability, opportunity, and motivation interact with behavior in a system [61]. The interviews will probe the capacity to engage in the study activities (capability), habits and decision-making around study involvement (motivation), external factors that influenced behavior, and completion of the study tasks (opportunity). Interview transcripts will be coded and organized into themes using NVivo (version 20; Lumivero; or newer) software. We will use an inductive approach to thematic analysis and aim to identify semantic and latent themes [62,63].

Where appropriate and scientifically sound, we will incorporate feedback on acceptability and feasibility to improve the study design. Changes to the study design due to feasibility or acceptability or based on feedback from prospective or enrolled participants will be documented.

#### Subgroup Analyses

We will compare acceptability and feasibility outcomes between study arms (AD, LBD, or controls) and conduct exploratory analyses to determine whether subjective sleep quality support from a relative or friend or baseline apathy and anxiety predict overall adherence to study tasks.

### Ethical Considerations

This study has been approved by the Health Research Authority (Yorkshire and the Humber—Bradford Leeds Research Ethics Committee, reference 21/YH/0177) and carries minimal risk to participants. The study will be conducted in accordance with Good Clinical Practice and the Helsinki Declaration to protect the rights and welfare of all participants. All data will be kept securely and handled in accordance with the General Data Protection Regulation (EU 2016/679). Capacity to consent to the research study will be assessed and participants will be required to provide full written informed consent prior to participation in any study activities. The participants will be reminded of their right to withdraw at any point, without providing a reason, and without this affecting their health care. Participants will be offered cash reimbursement for travel or postage expenses incurred during the study. The participants will be assigned a study ID at consent to allow pseudonymization of participant data, with personal information stored separately and securely from deidentified data. The final paper and any data shared will contain no information that allows for the identification of individual participants.

Results will be presented at scientific meetings and conferences and published in peer-reviewed journals. Summaries will be provided to participants where they have indicated consent to be contacted about results from the study.

## Results

The study opened to recruitment in February 2022. Participant recruitment is scheduled to be completed in 2023. Data collection is anticipated to continue until February 2024, with analysis beginning in 2023 and continuing into 2024.

Results will be reported in line with guidance from the STROBE checklist [64] and the CONSORT 2010 extension [65] for pilot and feasibility trials [66].

## Discussion

### Principal Findings

Sleep is a fundamental component of health, and sleep disturbances are commonly observed in people living with MCI and dementia. Insufficient or poor sleep may represent both a risk factor and a symptom of these conditions, but more work is needed to confirm the relationship between sleep and MCI or dementia. Improvements to the way we measure sleep, such as measuring sleep in someone’s natural home environment and using technologies to supplement data collected from sleep questionnaires may help us to better understand the sleep profile in these conditions compared to normal aging, identify targets for intervention, and monitor disease progression [32,67]. However, we first need to understand whether it is possible to collect good-quality sleep and cognitive data from people living with MCI and dementia in their own homes. Accordingly, this paper proposes a study to investigate the feasibility and acceptability of remote sleep and memory data collection using study tasks designed to be completed at home by people living with MCI or dementia. The study aims to assess whether people with MCI or dementia are willing to engage in sleep studies using technology and home-based study tasks, describe participant experience, and evaluate the study tasks based on retrieving complete and analyzable data. The findings from this study will guide future research design in sleep and memory.

### Limitations

The eligibility criteria for the study require participants to have an internet connection and be willing to use technology for the duration of the study. Understanding the feasibility and acceptability of technology-supported remote research is the purpose of the study; however, this inherently may introduce bias into the study. For example, it is possible this may mean those who are unfamiliar or uncomfortable with technology may not take part, or those who are particularly interested in technology may find the study more acceptable or feasible. If recruitment, retention rates, or feedback from patients who would otherwise be eligible but were unable or unwilling to take part because of limited access to or ability to use the internet or a smart device, we may offer an adapted version of the study and will record whether this influences recruitment rate and other relevant outcomes.

Subjective measures used in the study may be prone to recall bias and may be difficult for individuals with cognitive impairment to answer accurately. While participants themselves will need to provide answers to questions during the study, they can be supported by a family member, friend, or caregiver where needed and requested by the participant. Subjective measures will also be complemented by objective measures which are not prone to recall bias.

The longitudinal and remote nature of the study may result in a greater proportion of missing data compared to sleep research studies conducted in a laboratory setting. As a study enrolling persons with MCI and dementia, it is also expected that participants may have difficulty remembering to complete study tasks. Reasonable efforts will be taken to encourage adherence and data completion throughout the study period, particularly in the intensive week (eg, schedules and digital reminders via SMS text messaging or email). Automated scheduled reminders and task lists will be supplemented with ad hoc contacts from the research team directly (eg, contacting to ascertain reasons behind consecutive days of missing data).

### Conclusions

Technological advancements and improved digital literacy offer the opportunity to research sleep longitudinally and in the home environment. However, further research is needed to understand whether these developments may benefit MCI and dementia study design. This protocol outlines a mixed methods study that examines the feasibility and acceptability of remote sleep and cognitive testing in a cohort of older adults specifically those with MCI or dementia due to probable AD or LBD. Outputs from the study will inform the approach to studying sleep in people with MCI or dementia in this population, contributing toward global efforts to identify and better understand potentially modifiable risk factors in these conditions.

## Abbreviations

AD: Alzheimer disease
COM-B: Capability Opportunity Motivation-Behavior
CONSORT: Consolidated Standards of Reporting Trials
EEG: electroencephalography
LBD: Lewy body disease
MCI: mild cognitive impairment
MoCA: Montreal Cognitive Assessment
NHS: National Health Service
OSA: obstructive sleep apnea
PSG: polysomnography
REM: rapid eye movement
RESTED: Remote Evaluation of Sleep to Enhance Understanding of Early Dementia
STOP: Snoring Tiredness Observed Pressure
STROBE: Strengthening the Reporting of Observational Studies in Epidemiology

## Data Availability

Data sets generated during this study are available from the corresponding author upon reasonable request.
